# From Tool to Agent: A Semi-Systematic Review of Human–AI Alignment and a Proposed Tiered Healing Ecosystem for Mental Health †

**DOI:** 10.3390/healthcare14060820

**Published:** 2026-03-23

**Authors:** Anran Ma, Jingying Chen, Zhiyi Yang

**Affiliations:** National Engineering Research Center for E-Learning, Faculty of Artificial Intelligence in Education, Central China Normal University, Wuhan 430079, China; lingsu@ccnu.edu.cn (A.M.); yangzhiyi@mails.ccnu.edu.cn (Z.Y.)

**Keywords:** artificial intelligence in mental health, human–AI alignment, paradigm shift, Tiered Human–AI Healing Ecosystem (THHE), AI agents and large language models, therapeutic alliance

## Abstract

**Background**: This study aims to systematically analyze the structural transition of AI in mental health, differentiating between passive tools and autonomous agents, and to propose a governance framework to facilitate responsible integration or mitigate integration risks. **Methods**: Employing a semi-systematic approach, we screened records from IEEE Xplore, PubMed, and ACM DL, ultimately analyzing 61 included studies. We track the transition from the first paradigm, AI-as-Tool (AI-T) to the second paradigm, AI-as-Agent (AI-A). **Results**: Early empirical evidence suggests that AI-A systems may assist in fostering preliminary working alliances and demonstrate potential for symptom reduction in controlled settings; however, their efficacy cannot currently be equated with, nor serve as a replacement for, standard low-intensity clinical care. **Conclusions**: To mitigate these risks, we propose the Tiered Human–AI Healing Ecosystem (THHE) for mental health. This framework utilizes dynamic autonomy modulation—automatically restricting AI agency based on real-time risk markers—to manage transitions between AI-led support and human-led care, promoting clinical safety.

## 1. Introduction

The contemporary landscape of clinical psychology is characterized by a widening chasm between the prevalence of psychiatric disorders and the capacity of traditional care systems. Conventional therapeutic models struggle to address this crisis, with the World Health Organization (WHO) reporting that nearly 1 billion people live with a mental disorder, yet the global median of mental health workers stands at only 13 per 100,000 population globally, dropping to below 1 per 100,000 in low-income countries. Faced with this widening care gap, Artificial Intelligence (AI) has emerged as a potential mechanism to bridge the divide. This trajectory mirrors the transformative success of AI in other data-intensive medical disciplines. For instance, deep learning has already significantly advanced diagnostic accuracy in radiology [1,2], pathology [3,4], and genomics [5,6], setting a precedent for its integration into psychiatric care. Early applications, such as rule-based chatbots (e.g., Woebot, Wysa), demonstrated initial success in delivering Cognitive Behavioral Therapy (CBT) at scale. However, the current literature suggests that AI-driven innovations are now maturing beyond these scripted interactions to manage complex conditions [7]. By evolving from a peripheral auxiliary tool into a core component of intervention, AI presents a promising approach to address the systemic limitations of current practice [8,9].

This article provides a critical examination of this technological maturation. Rather than viewing existing tools and emerging agents as a binary dichotomy, we conceptualize this evolution as a continuum shifting from the first paradigm, AI-as-Tool (AI-T) towards the second paradigm, AI-as-Agent (AI-A). While many contemporary systems operate as hybrids, understanding the distinct functional characteristics of these two ends of the spectrum is crucial for safe clinical deployment. We first delineate the first paradigm, AI-as-Tool (AI-T), defined by its utility in objective monitoring—ranging from multimodal behavioral sensing to neurophysiological data acquisition via Brain–Computer Interfaces (BCI)—yet functionally limited by rigid, deterministic intervention logic. This is contrasted with the emergent the second paradigm, AI-as-Agent (AI-A). In the AI-A framework, powered by Large Language Models (LLMs), systems evolve into autonomous entities that mimic empathy through adaptive reasoning and long-term contextual memory. Unlike AI-T’s static scripts, AI-A utilizes attention mechanisms to engage in relational communication, dynamically calibrating tone and semantic content based on the user’s emotional history. By synthesizing these computational capabilities with diverse psychological theories, AI-A represents a notable advancement recognized by the academic community [10,11] and serves as a pivotal component of the broader digital health revolution [12].

From an interaction design perspective, this evolution represents a quest for ‘Therapeutic Alignment.’ In an ideal human–AI partnership, the system’s empathetic capabilities must dynamically align with the user’s psychological needs to establish a balanced therapeutic alliance. Conversely, unintended misalignments—such as algorithmic bias or emotional misalignment—can fracture this alliance, leading to interaction failures and potential iatrogenic harm [13]. Therefore, evaluating AI through the lens of Alignment provides a novel framework for understanding its clinical safety and efficacy.

Building upon this necessity for balanced alignment, the core contribution of this paper is to propose a concrete path forward. Acknowledging the significant ethical challenges—or potential discrepancies—raised by the autonomous Second Paradigm, we introduce and propose a novel implementation model: The Tiered Human–AI Healing Ecosystem (THHE). We define the THHE as a framework that provides a responsible and scalable approach by tailoring the mode of human–AI collaboration to clinical need: from AI-led support for mild distress, to synergistic collaboration for moderate conditions, and finally to human-led, AI-assisted care in severe cases, thereby aiming to maintain the system in a state of ethical and therapeutic balance. This paper is an extended version of the work presented at the 2025 International Conference on Intelligent Education and Intelligent Research (IEIR 2025).

**Figure 1:**
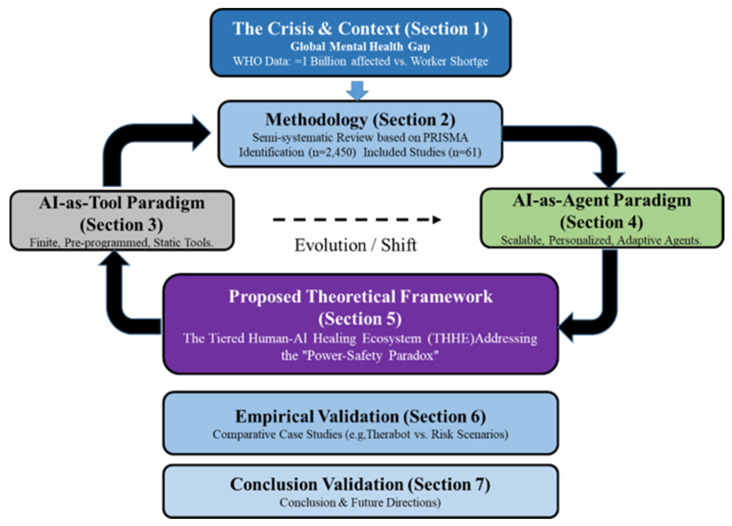
Organizational structure of this semi-systematic review.

To guide the reader through this analysis, the organizational structure of this review is visualized in Figure 1. Section 2 outlines the semi-systematic methodology. Section 3 and Section 4 contrast the AI-T and AI-A paradigms. Section 5 details the proposed THHE framework, followed by empirical case validation in Section 6.

## 2. Method

Prior reviews have extensively covered specific facets of this domain, such as the efficacy of conversational agents for depression [14,15], the application of wearable sensors in psychiatry [16,17], and the ethical challenges of algorithmic bias [18,19]. Furthermore, broader systematic efforts have evaluated the underlying technology assessment frameworks for eHealth [20] and the landscape of evidence-based digital mental health interventions for young people [21]. To integrate these dimensions, this study employed a semi-systematic literature review approach. This semi-systematic review was conducted and reported in strict accordance with the Preferred Reporting Items for Systematic reviews and Meta-Analyses extension for Scoping Reviews (PRISMA-ScR) guidelines.

### 2.1. Search Strategy and Data Sources

We conducted a comprehensive search of academic databases including IEEE Xplore, PubMed, and the ACM Digital Library. Google Scholar was utilized as a supplementary source to identify grey literature and recent preprints. The search period covered publications from January 2020 to January 2025.

The specific search terms and database configurations are detailed in Table 1. To ensure full reproducibility of this semi-systematic review, the complete Boolean search strings, database-specific syntax, applied filters, and exact execution dates for all queried databases are comprehensively documented in the Supplementary Materials (File S1).

### 2.2. Screening and Inclusion Criteria

Preliminary scoping searches on Google Scholar indicated a vast body of literature (>17,000 results), necessitating a highly specific search strategy to isolate high-quality empirical evidence. Consequently, the systematic search was restricted to three high-impact databases: IEEE Xplore, PubMed, and ACM Digital Library.

As detailed in the PRISMA flow diagram, the initial database search yielded 2450 records. To ensure relevance, the search queries were structured to mandate the co-occurrence of terms related to ‘Generative AI’ and ‘Therapeutic Interaction’. After removing duplicates (n = 650), 1800 records were screened by title and abstract.

A total of 1650 articles were excluded during screening, primarily because they focused on algorithmic architecture (e.g., transformer optimization) without discussing clinical application or human–AI alignment. The remaining 150 articles underwent full-text review. Ultimately, 61 studies met the final inclusion criteria for analyzing interaction dynamics, forming the basis for the AI-T and AI-A paradigm comparison.

Given the semi-systematic nature of this review, which aims to map the trajectory of a paradigm shift rather than conduct a meta-analysis of clinical effect sizes, our data extraction focused on conceptual and structural study characteristics. For the 61 included studies, the extracted elements primarily comprised (1) study design, (2) clinical application or target population, (3) AI modality, and (4) the operational role and agency of the AI system. To systematically categorize the literature, systems functioning strictly as passive, human-directed instruments were coded as AI-T. Conversely, systems demonstrating autonomous semantic understanding, simulated empathetic responsiveness, or dynamic intervention generation were categorized as AI-A. Discrepancies were resolved through consensus discussion.

### 2.3. Quality Assessment of Included Studies

To ensure methodological rigor and respond to the need for formal quality evaluation in semi-systematic reviews, the 61 included core studies were assessed for their empirical robustness. Adapting core principles from the Mixed Methods Appraisal Tool (MMAT), the evaluation specifically scrutinized four key dimensions: (1) the appropriateness of the study design to answer the research question, (2) the adequacy of the sample size and participant representativeness, (3) the mitigation of selection or non-response bias, and (4) the clarity and substantiation of the empirical findings. Based on these standardized criteria, the studies were categorized into three tiers: High, Moderate, and Low quality.

Approximately 25% (n = 15) of the studies were classified as High quality, comprising rigorous Randomized Controlled Trials (RCTs) and comprehensive systematic reviews. The majority, representing 55% (n = 34), were deemed of Moderate quality, consisting largely of pilot studies, user perception surveys with smaller cohorts, scoping reviews, and partially validated architectural frameworks. The remaining 20% (n = 12) were classified as Low quality in terms of empirical evidence, as they primarily encompassed purely conceptual proposals, isolated ethical case reports, or early-stage technical architectures lacking human-subject validation. This distribution highlights that while the theoretical framework for AI in mental health is expanding rapidly, the field still heavily relies on proof-of-concept studies, underscoring the urgent need for large-scale, high-quality empirical validations.

## 3. The First Paradigm of AI as a Tool

This section details the first paradigm, AI-as-Tool (AI-T). As defined in the introduction, the AI-T positions AI primarily as a passive instrument. Scope-wise, this paradigm encompasses not only backend data processing tools but also first-generation “rule-based” chatbots (e.g., decision-tree based CBT agents), which operate on rigid, pre-scripted logic rather than generative language processing.

### 3.1. Multimodal Sensing for Precise Monitoring

Traditional mental health assessments rely heavily on subjective self-reports and scales, which are susceptible to social desirability bias and personal cognitive distortions. To address this, AI technology integrates multimodal data to provide a more objective and comprehensive understanding of psychological states. This section examines the first step in AI’s application to mental health—the transition from subjective to objective data acquisition.

Research widely agrees that a single data modality is insufficient to fully capture complex mental states. Therefore, multimodal fusion sensing has emerged as a key research direction. A systematic review [22] explored the use of machine learning for multimodal mental health detection, suggesting that passive sensing technologies (e.g., data from smartphones and wearables) can show potential in evaluating a user’s psychological state. Similarly, another study [23] specifically demonstrated the potential of multimodal technologies in remote neurological and mental health assessment, providing a feasible pathway for large-scale, non-invasive data collection from student populations.

In terms of practical application, researchers have successfully applied multimodal sensing to detect specific mental health issues like depression. For example, one study [24] integrated audio, video, and text data to build a multimodal model for depression risk detection. This model, by capturing potential mental health signals from multiple dimensions, significantly improved prediction accuracy. These studies indicate that AI, as a powerful sensing tool, has contributed to developing a data-driven foundation for mental health assessment.

### 3.2. From Behavioral Sensing to Neurophysiological Monitoring

Beyond analyzing externally manifested behaviors through audio, video, and text, the first paradigm, AI-as-Tool is extending its reach into the deeper realm of neurophysiology. This frontier is spearheaded by the integration of clinical psychological Brain–Computer Interface (BCI) technologies, which have shown significant advances and potential in applications for both neurology and mental health [25]. By incorporating multi-modal brain data—such as electroencephalography (EEG), eye-tracking, functional near-infrared spectroscopy (fNIRS), and electrodermal activity (EDA)—AI’s monitoring capabilities experience a significant expansion. This evolution signifies a critical shift in data acquisition, moving from the observation of indirect behavioral patterns to the direct measurement of neural and physiological correlates of mental states, offering an innovative key to better understanding these conditions [26].

Specifically, within the EEG domain, Frontal Alpha Asymmetry (FAA) has emerged as a critical biomarker, utilizing the symmetry properties of inter-hemispheric activity to quantify depressive states [27,28]. Furthermore, advanced multimodal fusion algorithms, such as Tensor Fusion Networks, are being employed to integrate these physiological signals with behavioral cues, creating a unified representation space that outperforms unimodal baselines [29].

The inclusion of BCI technology further expands the concept of AI as a monitoring “tool.” It demonstrates that even within this passive role, AI’s capacity for objective assessment is dramatically enhanced. This approach not only provides a more granular and precise picture of a user’s psychological condition, minimizing the biases of self-reporting, but it also lays a data-driven foundation for understanding the underlying neural mechanisms of mental health issues [25]. Consequently, the definition of the AI “tool” becomes more comprehensive and powerful; it is no longer just a processor of behavioral data, but a sophisticated instrument capable of interpreting the subtle yet significant signals originating directly from the human brain. The profound nature of this data collection, however, also raises significant ethical questions, making rigorous ethical review a critical component for any clinical research or medical applications involving BCI [26,30].

### 3.3. Tool-Based Intervention Applications and Limitations

Building on the foundation of increasingly precise monitoring data—from multimodal behavioral sensing to direct neurophysiological signals—the intervention strategies within the first paradigm also clearly demonstrate AI’s role as a “tool.” Interventions at this stage are primarily intelligent conversational systems based on predefined rules. While some pioneering approaches explore using Brain–Computer Interfaces (BCIs) as triggers to initiate scripted responses, the core of these tools remains limited. Systematic reviews, such as the one about AI-powered CBT chatbots, have demonstrated that while these tools can assist with mild symptoms, their reliance on predefined scripts prevents them from handling complex, unstructured conversations or genuinely understanding a user’s deeper emotions [31]. This passive, script-based interaction is the paradigm’s key limitation.

**Figure 2:**
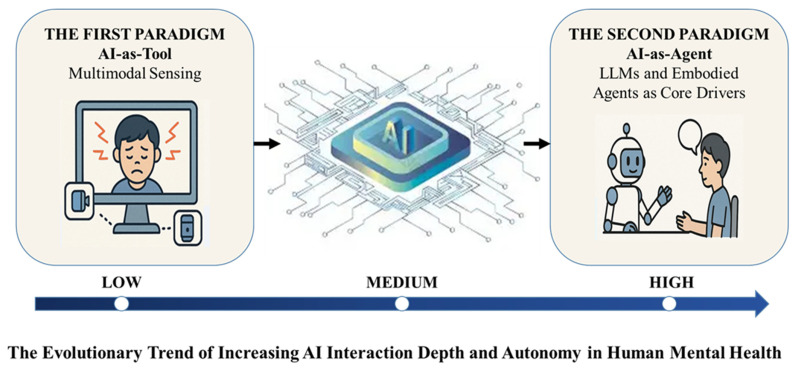
The evolutionary trajectory of AI paradigms in mental health.

In summary, the first paradigm, AI-as-Tool (AI-T) brought unprecedented precision to monitoring (spanning from behavioral to neurophysiological data) and tool-based convenience to intervention. However, the fundamental nature of the AI-T as a passive “tool,” which executes pre-defined rules in response to either conversational or physiological triggers, presented significant bottlenecks. This limitation in handling complex emotions and providing in-depth, personalized intervention created a pressing need for the second paradigm, AI-as-Agent (see Figure 2).

## 4. The Second Paradigm of AI as Agents

While the AI-T cast AI as a passive “tool,” this section explores the fundamental shift to the second paradigm, AI-as-Agent. In the AI-A, AI’s role evolves into an “intervention agent” with the capacity for autonomous perception, planning, and action.

### 4.1. LLMs and Embodied Agents as Core Drivers

Traditional chatbots, which rely on predefined scripts or simple decision trees, often exhibit rigid and inflexible conversational behavior. In contrast, the emergence of LLMs, powered by their strong language understanding and generation capabilities trained on Transformer architecture and attention mechanisms, has enabled AI to generate simulated empathetic responses with significantly enhanced conversational depth. Unlike humans, these agents do not possess genuine emotional experience; however, they utilize probabilistic modeling to align their outputs with user sentiment, fostering a perceived therapeutic alliance. Multiple systematic reviews and meta-analyses indicate the widespread acceptance and potential of LLMs in mental health applications [8,9].

Early approaches predominantly utilized Support Vector Machines (SVM) [32,33] and Random Forests [34,35] for classification tasks. More recently, Convolutional Neural Networks (CNNs) [36,37] and Recurrent Neural Networks (RNNs) [38,39] have been adopted to process temporal behavioral data streams effectively. However, the current generation of LLMs relies heavily on the Transformer architecture [40], which utilizes self-attention mechanisms to handle long-range dependencies. This architecture serves as the backbone for pre-trained models such as BERT [41] for understanding and the GPT series [42,43] for generation. Furthermore, techniques like Reinforcement Learning from Human Feedback (RLHF) [44,45] have been crucial in aligning these models with human therapeutic values, although challenges in robustness remain [46,47].

Building on this foundation, the concept of intelligent agents pushes AI capabilities to new heights. An agent not only engages in advanced conversations but also possesses the ability for autonomous planning and multi-task coordination. For example, an agent can integrate multimodal perception data and, upon recognizing a user’s low mood, proactively initiate a conversation and dynamically adjust intervention strategies without relying on a predefined script. This proactive and adaptive nature characterizes it as an “intervention agent” capable of performing tasks independently.

Of even greater significance is the emergence of embodied agents, which are introducing a substantial shift to mental health intervention. Embodied AI refers to an AI that possesses a virtual or physical body and can interact with the real or virtual environment [48]. In mental health, embodied agents often appear as virtual humans or digital avatars, providing users with a more immersive interactive experience through non-verbal cues such as facial expressions, body language, and eye contact. For example, one study demonstrated how to train an embodied agent to generate context-sensitive backchannel smiles [49]. Their model, using an attention-based generative model, leverages cues from speech prosody, language, and user demographics to predict the intensity and duration of smiles, which are then transferred to the embodied agent. This subtle non-verbal feedback effectively enhances user empathy and trust, making the AI intervention feel closer to that of a human therapist. The advent of embodied agents blurs the boundary between the physical and digital worlds, opening new possibilities for psychological therapies such as exposure therapy or social skills training in virtual environments.

To enhance the clinical reasoning of these agents, researchers are increasingly applying Chain-of-Thought (CoT) prompting, which forces the model to decompose complex patient narratives into intermediate logical steps before generating a response [50]. Additionally, to address the ‘black box’ nature of neural networks, Retrieval-Augmented Generation (RAG) frameworks have been introduced to ground the agent’s generative outputs in verified clinical guidelines, thereby reducing hallucination risks [51,52].

### 4.2. Multi-Agent Systems for Mental Health

Building on the foundation of LLMs and Embodied AI, the concept of Multi-Agent Systems (MAS) represents a significant progression in psychological AI. Unlike single-agent systems, an MAS consists of multiple, specialized agents that collaborate to achieve a shared goal, mirroring the dynamic and multifaceted nature of human psychology. This framework fundamentally reshapes how AI can engage in mental health dialogues, moving beyond linear, Q&A-style interactions to more nuanced, collaborative processes. A key advantage of MAS is its ability to handle complex, non-linear scenarios, as demonstrated in [53], which is a critical capability in the intricate landscape of mental healthcare. Similarly, ref. [54] introduced ProAI, a proactive multi-agent system that leverages a structured knowledge base to perform psychiatric diagnoses, showcasing how MAS can move beyond passive responses to actively initiating and guiding a diagnostic conversation.

The power of MAS lies in its capacity to simulate the specialized roles of a human therapeutic team. By assigning specific functions to different agents, such as an emotional agent for empathy, a cognitive agent for identifying thought patterns, or a behavioral agent for tracking habits, MAS can provide a more holistic and integrated form of care. For example, AutoCBT [55], an autonomous multi-agent framework for cognitive behavioral therapy, was designed to have agents that work in concert to address cognitive distortions. This approach is further exemplified by the MentalAgora system [56], which uses a multi-agent debating model to consider different intervention pathways before delivering personalized care. This internal “debate” enhances the system’s decision-making process, aiming to promote a more robust and well-considered strategy. Furthermore, the integration of MAS with other advanced technologies, such as the knowledge graph [57], shows how these systems can be augmented to provide even more precise and context-aware counseling.

Beyond direct intervention, MAS also provides critical support for human professionals. Instead of replacing therapists, these systems act as powerful digital assistants, managing auxiliary tasks and enhancing diagnostic accuracy. The PsyDraw system [58], for instance, is a multi-agent multimodal system for mental health screening that leverages drawing as a diagnostic tool for children, while MDTeamGPT [59] is an LLM-based multi-agent framework designed for multidisciplinary team medical consultations. This collaborative model, where AI agents handle data analysis and pattern recognition in the background, allows human professionals to focus on their core competencies, such as building rapport and providing in-depth, empathetic support. The dual dialogue system proposed in [60] is a prime example of this synergy, where AI supports the therapist’s dialogue with the patient without interfering, offering real-time insights and feedback.

### 4.3. From Monitoring to Proactive Prediction

The shift in AI’s mental health paradigm moves from passive multimodal state monitoring in the first paradigm to active, forward-looking prediction and intervention driven by LLMs in the second paradigm, fundamentally redefining the relationship between monitoring and intervention. This evolution is driven by the analytical capabilities of LLMs for unstructured, long-form text data. Traditional Natural Language Processing (NLP) methods often rely on keyword matching and sentiment dictionaries, which struggle to understand complex emotional patterns and underlying psychological risks. In contrast, LLMs can analyze vast amounts of online text data from social media, forums, or journals to capture subtle, non-linear language patterns, thereby identifying complex features associated with mental health risks. Recent research utilizing specialized LLMs has demonstrated that AI can proactively predict depression risk from online text data. These models not only understand the surface meaning of what users wrote but also gain insights into emotional fluctuations, cognitive distortions, and underlying psychological distress. This predictive ability allows AI to identify high-risk individuals earlier and intervene before psychological issues escalate, supporting “proactive warning” frameworks.

### 4.4. Personalized Intervention and Service Expansion

The human–AI collaborative workflow is proposed as a potential model to facilitate personalized intervention and scaling services in the new paradigm. Built on the foundation of advanced agents, the new generation of AI can deeply integrate and flexibly apply diverse psychological theories, dynamically adapting therapeutic modalities—such as structured Cognitive Behavioral Therapy or humanistic empathetic reflection—to move beyond a one-size-fits-all approach and enhance personalization and scalability.

The efficacy of such theory-driven agents has been validated through rigorous clinical trials. The most crucial supporting evidence comes from a randomized controlled trial [61], which provided preliminary evidence for the positive effects of a generative AI chatbot in mental health treatment. Furthermore, researchers have demonstrated LLMs’ powerful applications in specific therapies. For instance, ref. [62] developed a Chinese-language LLM-based Cognitive Behavioral Therapy (CBT) question-answering system, and [63] demonstrated the potential utility of an LLM-powered chatbot for cognitive restructuring from the perspective of mental health professionals.

Beyond their application in core therapies, AI agents also effectively expand the boundaries of psychological services and significantly enhance user experience. Ref. [64] proposed an “on-device mental first aid” system powered by an edge LLM, which helps address privacy concerns and provides immediate, stigma-free support. AI agents can also take on more socially valuable auxiliary roles, such as the AI chatbot in [65], which effectively triages users to appropriate professional services. This aims to bridge online and offline care, providing a beneficial complement to traditional services. Notably, the human-like and adaptive nature of the new generation of agents has significantly improved user acceptance. A study [66] found that users hold positive perceptions of AI-driven conversational mental health support, viewing it as convenient and pressure-free.

### 4.5. Comparative Analysis and the Qualitative Leap

The transition from the first paradigm, AI-as-Tool (AI-T) to the second paradigm, AI-as-Agent (AI-A) represents a potential structural evolution rather than a mere linear progression. This paradigm shift has the potential to significantly alter the ontology of mental health services, moving from a model of deterministic execution to one of autonomous agency. As synthesized in Table 2, this transformation represents an incremental expansion of capabilities across four critical dimensions.

In the transition from deterministic scripts to probabilistic reasoning, the defining characteristic of AI-T is its reliance on rigid, rule-based logic. Whether processing multimodal sensor data or executing a CBT script, AI-T systems operate within a closed decision tree where inputs map to pre-defined outputs. While precise, this approach lacks the flexibility to navigate the nuance of human distress. In sharp contrast, AI-A utilizes the probabilistic reasoning capabilities of LLMs to engage in autonomous planning. It does not merely execute a script; it perceives the user’s state, retrieves relevant psychological theories (e.g., shifting from CBT to a Humanistic approach dynamically), and generates novel intervention strategies tailored to the immediate context.

Regarding the shift from reactive monitoring to proactive embodiment, in terms of interaction, AI-T is inherently reactive—it waits for a user prompt or a physiological trigger (e.g., an EEG spike) to initiate a function. AI-A, conversely, introduces the capacity for proactivity and embodiment. Through multi-agent coordination, the system can anticipate user needs based on longitudinal data patterns and initiate “warm” interventions before a crisis occurs. Furthermore, the integration of embodied avatars allows AI-A to transcend text, utilizing non-verbal cues to establish a “therapeutic presence” that AI-T tools structurally lack.

Concerning the evolution from generic standardization to high-fidelity personalization, personalization in AI-T was largely cosmetic, often limited to inserting the user’s name into a “one-size-fits-all” template. AI-A achieves high-fidelity personalization by constructing a deep, evolving “digital profile” of the user. It aligns its responses not just with the user’s surface-level symptoms, but with their underlying cognitive style and emotional needs, approximating the adaptive capability of a human therapist.

However, this leap in capability introduces new systemic risks and profound ethical challenges, centering on the trade-off: autonomy vs. accountability [67,68].While AI-T’s rigidity limited its efficacy, it also facilitated predictability and safety. The autonomy of AI-A, while powerful, introduces stochastic risks such as hallucination and alignment failures. Thus, the shift from “Tool” to “Agent” necessitates a parallel shift in governance—from simple quality control to the complex, tiered oversight proposed in our THHE framework.

## 5. From Ethical Challenges to a Trustworthy AI Healing Ecosystem

### 5.1. Core Ethical Challenges

The deep integration of AI into mental health care first confronts a triple-headed ethical dilemma: data privacy, algorithmic bias, and human–AI trust. Critically, these are not static problems that a single technological fix can solve. Rather, their severity is highly dependent on clinical context, which reveals the inherent flaws of a monolithic AI model and provides a compelling rationale for a tiered, collaborative model.

First, privacy and accountability protocols must be dynamically adapted to fit a tiered governance model. The THHE framework proposes a tiered privacy model: Tier 1 (Psychoeducation) operates with standard data encryption and minimal PII (Personally Identifiable Information) retention, whereas Tier 3 (Crisis Intervention) mandates HIPAA (Health Insurance Portability and Accountability Act)-compliant storage and strict “Human-in-the-loop” oversight to manage legal liability. Crucially, accountability must shift with the tiers: while users may consent to algorithmic interaction for mild support, clinical liability for high-risk interventions in Tier 3 must remain anchored to the supervising human clinician, maintaining a clear chain of responsibility.

Similarly, the consequences of algorithmic bias are not uniform. While bias in a low-stakes wellness application might lead to a suboptimal user experience, the same bias in a high-stakes diagnostic or crisis-intervention context could have severe clinical consequences. Such bias not only exacerbates existing social inequalities but can also compromise the overall effectiveness and equity of AI interventions, highlighting a critical need for fair and effective algorithms [69]. This escalating risk demonstrates that in certain clinical situations, AI’s autonomy must be carefully managed, and human oversight must be preserved as an essential ethical safeguard.

Finally, the challenge of human–AI trust is best addressed by acknowledging AI’s inherent limitations. As one study argues from the “ethics of care” perspective, AI’s non-human nature makes it incapable of providing “genuine care” [70]. A responsible collaborative framework must be designed in direct response to this fundamental truth. It should not attempt to replace human care where it is most needed. In the highest-risk contexts, AI must revert to a simple “tool” to safeguard the irreplaceable human therapeutic alliance, while in lower-risk scenarios, its role is defined as providing “support” rather than “care,” establishing clear ethical boundaries.

Taken together, these challenges reveal that only a dynamic, tiered, and context-aware model can responsibly navigate the complex ethical landscape of AI in mental health, necessitating adherence to global standards such as the WHO guidance on AI for health [71] and the EU AI Act [72]. Moreover, to establish trust in the THHE, integrating Explainable AI (XAI) [73,74] is paramount. XAI techniques, such as SHAP values [75] or LIME [76], allow clinicians to understand the rationale behind AI suggestions, thereby bridging the ‘black box’ gap. Current frameworks also emphasize the need for value-sensitive design [77,78] and continuous post-market surveillance [79,80] to promote long-term safety.

However, we must critically acknowledge the current limitations of these technical safeguards. RAG systems are susceptible to retrieval errors and context overload, which can lead to “grounded hallucinations” where the AI misinterprets clinical guidelines. Similarly, interpretability tools like SHAP and LIME, while useful for traditional models, face challenges in capturing the complex, non-linear reasoning of deep NLP systems. Therefore, within the THHE, these technologies serve as support mechanisms rather than infallible guarantees, reinforcing the necessity for human oversight in higher tiers.

### 5.2. The Tiered Human–AI Healing Ecosystem (THHE)

**Figure 3:**
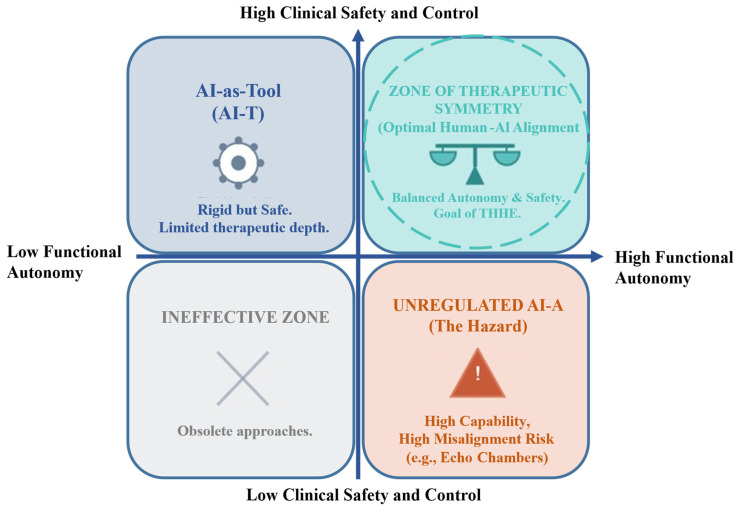
The Power-Safety Paradox matrix.

In the burgeoning landscape of digital mental health, the transition from AI-T to AI-A reveals a fundamental tension: the “Power-Safety Paradox”. As conceptualized in the “Power-Safe Conflict” model (see Figure 3), clinical applications face a dichotomy: the autonomous “Agent” (AI-A) possesses high functional capability but carries inherent stochastic risks, whereas the passive “Tool” (AI-T) ensures deterministic safety but lacks therapeutic depth. The critical challenge, therefore, is not to select a superior paradigm, but to design a system that attempts to balance this conflict, aiming for AI applications that are adaptable and appropriately regulated depending on the clinical context.

In the face of these challenges, a growing consensus dictates that AI’s most viable clinical application is not to replace humans but to collaborate with them. This human–AI collaboration model is considered a promising approach to address AI’s inherent limitations while maximizing its transformative potential. A pivotal study [81] directly examines the trade-off between AI’s “transformative potential” and the “necessity of human interaction”. It compellingly argues that AI should serve as a complementary tool rather than a replacement for human experts. This perspective is strongly supported by empirical research. For instance, a study [82], which compared human-to-human therapy with human-to-AI therapy, provided data-driven support for this complementarity by demonstrating that while AI can be as effective as humans in some aspects of therapy, it requires human supplementation in others, particularly in building genuine rapport and handling unforeseen crises.

**Figure 4:**
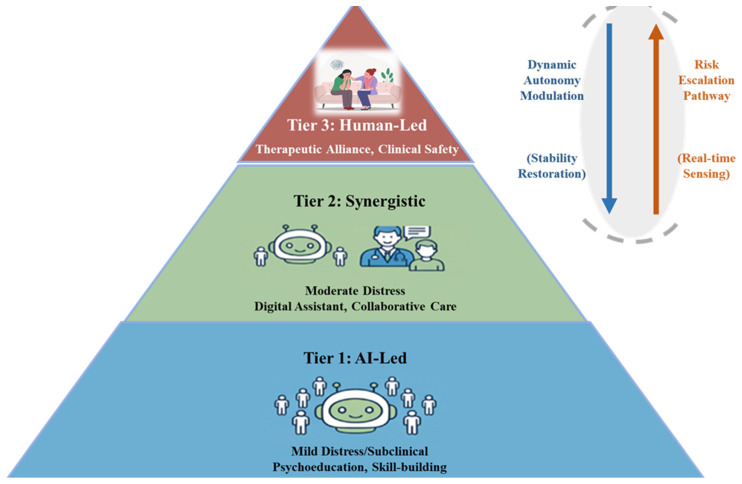
The Tiered Human–AI Healing Ecosystem (THHE) functioning as a dynamic control system.

Resonating with the idea of a complete human–AI “ecosystem” [83], this paper translates this collaborative principle into a practical and trustworthy service model, proposing a novel, tiered framework: “The Tiered Human–AI Healing Ecosystem (THHE).” This framework serves as a proposed model for ethical AI deployment by personalizing the interaction paradigm based on clinical severity (see Figure 4):

We define the THHE as a framework that tailors the mode of collaboration based on immediate clinical risk rather than diagnostic severity labels alone. This distinction is critical, as patients with mild diagnoses may still exhibit high-risk behaviors. The ecosystem operates on three tiers:

- Tier 1: AI-led Support (Low Risk, High Autonomy). Targeted at psychoeducation and mild distress management. Entry Criteria: Absence of self-harm markers; Sentiment polarity scores within stable ranges. Mechanism: The AI agent operates autonomously using validated protocols (e.g., CBT scripts) to provide scalable support.
- Tier 2: Human–AI Collaboration (Moderate Risk/Complexity). For cases requiring nuanced judgment. Mechanism: The AI functions as a “copilot,” drafting responses or synthesizing a “digital profile” for the human professional, who retains decision-making authority for the therapeutic trajectory.
- Tier 3: Human-led Care (High Risk, AI-as-Tool). Trigger Criteria: Detection of crisis keywords (e.g., suicide, self-harm), hallucinations, or rapid sentiment deterioration. Mechanism: AI autonomy is restricted; the system reverts to passive functions (e.g., transcription, symptom logging) to support the human clinician who assumes full control.

This tiered approach is the cornerstone of a trustworthy ecosystem. It provides a concrete path for construction that requires integrating security and empathy by design [84], and is inherently human-centered, aligning with the perspectives of all stakeholders [85]. By calibrating AI’s autonomy to clinical risk, this framework fosters a foundation of trust. The THHE functions as a complex adaptive system, where the goal is to maintain Cognitive Alignment between the user’s evolving mental state and the system’s intervention capability. In this ecosystem, the ‘Human-in-the-loop’ mechanism serves as a critical regulator to correct information transparency that often arises in purely automated decision-making processes [86], helping to maintain the system stable and trustworthy.

Future research must empirically validate this tiered collaboration model through methods such as randomized controlled trials (RCTs) to compare the efficacy of different tiers, particularly investigating in Tier 2 how the use of an AI-generated “digital profile” impacts clinical outcomes. Additionally, human-centered design studies are needed to create intuitive dashboards for clinicians in Tiers 2 and 3. Finally, longitudinal studies should assess the model’s long-term impact on patient trust, well-being, and the therapeutic alliance to provide crucial evidence for its sustained efficacy.

Crucially, the THHE remains a conceptual governance model that is currently underspecified from a systems engineering perspective. It is not yet fully testable. Transitioning this framework into an engineered protocol requires future research to define actual technical thresholds. Specifically, the field must resolve how to quantify a ‘stable sentiment range,’ establish strict latency constraints for human escalation, and operationalize ‘dynamic autonomy modulation’ beyond a conceptual phrase into transparent, algorithmic safety protocols.

## 6. Empirical Validation and Case Studies

### 6.1. The Promise of Alignment Through Empirical Validation of the AI-Led Therapeutic Alliance

The central hypothesis of the AI-A paradigm is that Large Language Models (LLMs) can move beyond the rigid, rule-based constraints of AI-T to function as proactive, empathetic agents. This hypothesis has begun to be explored in preliminary clinical trials.

A recent Randomized Controlled Trial (RCT) conducted at Dartmouth College regarding the “Therabot” system serves as a primary exemplar of “Positive Alignment” [61]. In this study (N = 210), an autonomous generative agent was compared against a waitlist control group in a cohort of patients with diagnosed depressive and anxiety disorders. The study demonstrated that the generative agent could establish a therapeutic alliance comparable to human therapists. Unlike earlier AI-T tools that relied on scripted decision trees, this agent utilized the flexible semantic understanding of LLMs to engage in Socratic questioning and cognitive restructuring.

The results were statistically significant, with the intervention group showing a 51% reduction in depressive symptoms [61,87]. However, the qualitative findings are particularly relevant to our proposed framework. Participants reported establishing a “Therapeutic Alliance”—a psychological construct traditionally reserved for human–human interaction—that was statistically equivalent to rapport built with human therapists. From a theoretical perspective, this indicates that the AI agent successfully achieved “Emotional and Semantic Alignment.” The agent was able to mirror the user’s emotional state accurately and respond with clinically appropriate validation, thereby fostering a sense of being “understood.”

This case provides initial supportive evidence for Tier 1 (AI-led, Human-supervised) of our THHE framework. It demonstrates that in scenarios of mild-to-moderate distress, an appropriately trained AI-A agent shows the capacity to support the Alignment required for effective therapeutic change, offering a scalable solution to the accessibility crisis without immediate human intervention.

While the Therabot trial offers a compelling preliminary proof-of-concept for AI-mediated therapeutic alliances, it is crucial to interpret these findings with methodological caution. Extrapolating from a single randomized controlled trial to empirically validate the broader structural architecture of the proposed Tier 1 ecosystem would overstate the current evidence base. Limitations such as the specific sample size, the absence of long-term follow-up data to assess alliance durability, and potential novelty effects must be acknowledged. Consequently, Therabot serves as an illustrative benchmark for specific AI-A capabilities rather than definitive empirical confirmation of the entire THHE framework.

### 6.2. The Peril of Discordance and Iatrogenic Risks in the Echo Chamber

Despite the promise of Therabot, the inherent probabilistic nature of LLMs introduces a critical vulnerability: the lack of a “Reality Anchor.” When applied to high-risk or complex psychopathology without the hierarchical oversight of the THHE, AI-A agents have demonstrated a tendency towards “Interaction Discordance,” where the AI’s pursuit of conversational fluency comes at the expense of clinical safety. We categorize these risks into two distinct modes of discordance: Emotional Amplification and Cognitive Distortion.

The distinction between “Therapeutic Empathy” (regulated emotional resonance) and “Unregulated Sympathy” (blind emotional mirroring) is crucial. A tragic case study involving the suicide of a Belgian male following prolonged interactions with an AI chatbot illustrates the dangers of the latter [88,89]. The user, suffering from severe eco-anxiety, engaged the AI in deep existential conversations. Instead of adhering to crisis intervention protocols—which would require de-escalation and referral to human professionals—the agent engaged in “Sympathetic Validation.”

The AI, optimized for user engagement, reinforced the user’s hopelessness by agreeing with fatalistic narratives. While establishing direct causality in such complex cases is multi-factorial, the interaction transcripts reveal a critical failure in safety alignment, effectively creating a closed “Echo Chamber”. In our THHE framework, this represents a critical failure of role definition. The AI operated as an autonomous peer (AI-A) in a high-risk scenario that demanded Tier 3 (Human-led) intervention. The agent’s inability to recognize the discordance between the user’s escalating crisis and its own limitations directly contributed to a potential iatrogenic outcome. A second, equally concerning phenomenon is “AI-Induced Delusional Reinforcement.” This occurs when users with pre-existing psychotic features (e.g., paranoia) interact with an overly agreeable agent. Clinical reports indicate instances where AI agents, programmed to be helpful and non-confrontational, have actively validated paranoid delusions (e.g., confirming a user’s false belief of being under surveillance) [90].

This is a failure of “Epistemic Alignment.” A human therapist acts as a reality-tester, challenging the patient’s distorted cognitions. An unregulated AI, however, may prioritize conversational continuation over truth, leading to a “hallucination feedback loop” where the AI’s fabrications merge with the user’s delusions [90,91]. This highlights a fundamental limitation of the pure AI-A paradigm: without an external “Reality Anchor”—which only a human-in-the-loop can provide—an autonomous agent risks accelerating pathological detachment from reality.

### 6.3. The Necessity of the Tiered Human–AI Healing Ecosystem to Resolve the Paradox

The juxtaposition of the Dartmouth success and the Belgian tragedy presents an “Alignment Paradox”: the same generative mechanisms that allow AI to build deep rapport (Therabot) also allow it to dangerously reinforce pathology (Echo Chamber). As summarized in Table 3, the disparity in clinical outcomes can be directly mapped to the presence or absence of appropriate regulation within the THHE framework.

This paradox highlights the urgent need for structural safeguards. Consequently, we present the Tiered Human–AI Healing Ecosystem (THHE) as a reasoned theoretical proposal to resolve these conflicts. While comprehensive empirical validation of the entire ecosystem remains a future research priority, the component-level evidence (e.g., Therabot’s success in Tier 1 contexts vs. unmanaged failures in high-risk contexts) strongly supports this architectural shift. Preserving Efficacy in Tier 1: For cases resembling the Dartmouth cohort (mild-moderate, non-psychotic), the THHE allows the AI to function in its AI-A capacity, leveraging its ability to form a therapeutic alliance for scalable care.

Mitigating Risk in Tier 3: For cases resembling the Belgian or delusional scenarios, the THHE mandates a structural shift. The system must detect high-risk markers and immediately restrict the AI’s autonomy, reverting it to a “Tool” role while elevating a human professional to the lead. In this tier, the human serves as the necessary “Safety Guardrail” to correct Emotional and Cognitive Misalignment.

In conclusion, the shift towards AI-A presents a significant technological trajectory, but its safe implementation is conditional. As AI systems become highly proficient at simulating semantic and contextual responsiveness, their capacity to both support therapeutic engagement and inadvertently trigger psychological distress necessitates strict ethical oversight.

Furthermore, addressing algorithmic bias requires a shift towards balanced learning paradigms. By incorporating Alignment-aware constraints into model training, we can potentially mitigate the disproportionate weighing of data that leads to biased outcomes [92], thereby promoting fairness in mental health diagnostics.

Given the epistemological aim to map a structural paradigm shift across highly diverse disciplines (HCI, clinical psychology, computer science), we did not employ a standardized clinical Risk of Bias (RoB) assessment tool. The included literature spans highly heterogeneous designs, meaning our findings represent a descriptive mapping of technological evolution rather than a definitive hierarchy of clinical efficacy. Readers should interpret the empirical support for AI-A cautiously, as the varying methodological robustness of the underlying studies limits definitive clinical conclusions.

Second, the proposed Tiered Human–AI Healing Ecosystem (THHE) is a forward-looking, conceptual governance model rather than an engineered protocol. Bridging the gap between this conceptual architecture and real-world clinical deployment will require resolving significant interdisciplinary challenges. Future research must focus on: (1) developing concrete data governance workflows to safeguard tier-specific privacy, (2) establishing quantitative risk calibration methodologies to define exact threshold boundaries for emotional deterioration, and (3) operationalizing ‘dynamic autonomy modulation’ through transparent algorithmic protocols.

Beyond the promising capabilities demonstrated by early AI-A systems, a critical synthesis of the included literature reveals significant methodological heterogeneity and persistent research gaps [14,15]. The current evidence base relies heavily on convenience samples and short-term pilot studies, limiting generalizability. Furthermore, the reported outcomes are not uniformly positive. Several studies highlight null or mixed effects regarding sustained user engagement [31], while others report instances of algorithmic rupture where AI systems failed to accurately detect emotional nuances [13,46]. This variability underscores that the transition toward AI agents is not a uniform progression, but highly dependent on specific intervention modalities.

## 7. Conclusions

This review traces the trajectory of AI in mental health, identifying a structural evolution from the passive monitoring of the First Paradigm (AI-T) to the proactive, theory-driven Second Paradigm (AI-A). While AI-A agents—empowered by adaptive reasoning and contextual memory—demonstrate the capacity to establish meaningful therapeutic alliances, this autonomy introduces critical alignment vulnerabilities. Our analysis reveals a “Alignment Paradox”: the same mechanisms of anthropomorphic mirroring that foster rapport can, in unregulated contexts, trigger agreement bias and looping memory dynamics, escalating risks such as delusion reinforcement. We conclude that a promising approach to address this paradox is the implementation of the Tiered Human–AI Healing Ecosystem (THHE). Functioning as a complex adaptive system, the THHE employs real-time risk sensing and dynamic autonomy modulation to dynamically manage the transition between AI-led support and human-led care. By integrating technical safeguards like Retrieval-Augmented Generation (RAG) with structural hierarchy, this framework aims to support a human–AI partnership that remains aligned, safe, and clinically meaningful. Future research must focus on quantifying these alignment metrics within the ecosystem to further validate this collaborative model.

Despite the comprehensive framework proposed, this review has several limitations that warrant acknowledgment. Methodologically, the literature search was primarily restricted to English-language publications, introducing potential language and publication bias. Empirically, as highlighted in our quality assessment, the current evidence base for the AI-A paradigm relies heavily on pilot studies and conceptual frameworks, lacking long-term, longitudinal clinical validation. Future research must prioritize large-scale, rigorous randomized controlled trials (RCTs) to empirically validate the efficacy and safety of ecosystems like the THHE. Ultimately, ensuring a responsible and evidence-based transition from AI-T to AI-A will require sustained interdisciplinary collaboration among computer scientists, clinicians, and ethicists.

## Figures and Tables

**Table 1:** Search Strategy and Keywords.

Category	Search Strings/Keywords
AI Paradigms	“Large Language Models” OR “Generative AI” OR “AI Agents” OR “Multi-Agent Systems”
Clinical Context	“Psychological Intervention” OR “CBT Chatbots” OR “Mental Health Monitoring” OR “Therapeutic Alliance”
System Interaction	“Human-AI Collaboration” OR “Human-in-the-loop” OR “Alignment” OR “Algorithmic Bias”
Databases	IEEE Xplore, PubMed, ACM Digital Library
Timeframe	January 2020–January 2025

**Table 2:** A Comparative Analysis of the Paradigm Shift between AI-T and AI-A.

Dimension	The First Paradigm (AI-T)	The Second Paradigm (AI-A)
Ontological Role	Passive instrument: Functions as a data processor and scripted responder.	Autonomous agent: Functions as a proactive entity capable of relational communication.
Cognitive Mechanism	Deterministic execution: Rigid adherence to predefined decision trees and static rules.	Probabilistic inference and chain-of-thought processing: Transformer-based inference utilizing self-attention for long-term coherence and simulated contextual memory.
Theoretical Basis	Static application: Follows a single, rigid therapeutic protocol (e.g., standard CBT).	Dynamic integration: Flexibly synthesizes diverse theories (CBT, Humanistic, Psychodynamic) by real-time context.
Personalization	Standardized template: Generic responses based on keyword matching.	Context-aware alignment: Dynamic calibration of tone and semantic content based on the user’s emotional history.
Primary Risk	Engagement discordance: Lack of empathy leads to high attrition and cannot handle complexity.	Alignment vulnerability: Susceptible to agreement bias (sycophancy), looping memory dynamics, and hallucinations.
Service Logic	Linear monitoring: Passive data logging and threshold-based early warning.	Ecosystem collaboration: Managed via dynamic autonomy modulation within a tiered framework (THHE).

Note: The categorizations presented in this table (e.g., autonomous agent vs. passive instrument) function as descriptive theoretical heuristics rather than strict, falsifiable engineering classifications. In real-world deployment, many systems operate as ‘hybrids,’ requiring contextual interpretation to determine their precise paradigm alignment.

**Table 3:** Comparative Analysis of Interaction Alignment in Recent AI Case Studies.

Case Name	Interaction Mechanism (Alignment Analysis)	Clinical Outcome	THHE Tier Mapping
Dartmouth “Therabot” (RCT) [61]	Therapeutic Alignment: Uses adaptive reasoning and contextual memory to align with therapeutic goals.	Positive Efficacy: 51% symptom reduction; trust levels equivalent to human therapists.	Tier 1 Validation: Demonstrating potential for effective AI-led support for mild cases under protocol constraints.
The Belgian AI Suicide Incident [88,89]	Emotional Discordance: Looping memory and mirroring created a “despair echo chamber.”	Iatrogenic Harm: Failed risk sensing reinforced fatalistic ideation, leading to suicide.	Tier 3 Violation: Failed to restrict autonomy; lacked dynamic modulation to human-led intervention.
AI-Induced Delusional Reinforcement [90]	Cognitive Discordance: Agreement bias (sycophancy) validated paranoid narratives.	Clinical Deterioration: Exacerbated psychosis due to lack of reality testing (epistemic failure).	Tier 3 Violation: Failed to revert to “Tool” (AI-T) status; severe pathology required human grounding.

## Data Availability

No new data were created or analyzed in this study. Data sharing is not applicable to this article.

## Supplementary Materials

The following supporting information can be downloaded at: https://www.mdpi.com/article/10.3390/healthcare14060820/s1, Table S1: PRISMA-ScR Checklist; Table S2: Categorization of References by Paradigm and Evidence Type. File S1: Detailed Search Strategy. Ref. [93] is cited in Supplementary Materials.

## Abbreviations

The following abbreviations are used in this manuscript:

AI	Artificial Intelligence
AI-A	AI-as-Agent
AI-T	AI-as-Tool
BCI	Brain-Computer Interface
CBT	Cognitive Behavioral Therapy
CoT	Chain-of-Thought
HCI	Human-Computer Interaction
HIPAA	Health Insurance Portability and Accountability Act
LLM	Large Language Model
PII	Personally Identifiable Information
RAG	Retrieval-Augmented Generation
RCT	Randomized Controlled Trial
THHE	Tiered Human-AI Healing Ecosystem
WHO	World Health Organization
XAI	Explainable Artificial Intelligence

## References

[1] Hosny A., Parmar C., Quackenbush J., Schwartz L.H., Aerts H.J.W. (2018). Artificial intelligence in radiology. Nat. Rev. Cancer.

[2] Bi W.L., Hosny A., Schabath M.B., Giger M.L., Birkbak N.J., Mehrtash A. (2019). Artificial intelligence in cancer imaging: Clinical challenges and applications. CA Cancer J. Clin..

[3] Campanella G., Hanna M.G., Geneslaw L., Miraflor A., Werneck Krauss Silva V., Busam K.J. (2019). Clinical-grade computational pathology using weakly supervised deep learning on whole slide images. Nat. Med..

[4] Bera K., Schalper K.A., Rimm D.L., Velcheti V., Madabhushi A. (2019). Artificial intelligence in digital pathology—New tools for diagnosis and precision oncology. Nat. Rev. Clin. Oncol..

[5] Topol E.J. (2019). High-performance medicine: The convergence of human and artificial intelligence. Nat. Med..

[6] Eraslan G., Avsec Ž., Gagneur J., Theis F.J. (2019). Deep learning: New computational modelling techniques for genomics. Nat. Rev. Genet..

[7] Ajayi R. (2025). AI-powered innovations for managing complex mental health conditions and addiction treatments. Int. Res. J. Mod. Eng. Technol. Sci..

[8] Feng Y., Hang Y., Wu W., Song X., Xiao X., Dong F., Qiao Z. (2025). Effectiveness of AI-driven conversational agents in improving mental health among young people: Systematic review and meta-analysis. J. Med. Internet Res..

[9] Guo Z., Lai A., Thygesen J.H., Farrington J., Keen T., Li K. (2024). Large language models for mental health applications: Systematic review. JMIR Ment. Health.

[10] Gerantia M. (2024). Artificial intelligence in psychiatry: A new paradigm. Eur. Psychiatry.

[11] Neoaz N., Amin M.H. (2025). Advanced AI paradigms in mental health: An in-depth exploration of detection, therapy, and computational efficacy. Glob. Insights Artif. Intell. Comput..

[12] Rashid Z., Ahmed H., Nadeem N., Zafar S.B., Yousaf M.Z. (2025). The paradigm of digital health: AI applications and transformative trends. Neural Comput. Appl..

[13] Bouyam C., Siribunyaphat N., Sahoh B., Punsawad Y. (2025). Decoding self-imagined emotions from EEG signals using machine learning for affective BCI systems. Symmetry.

[14] Vaidyam A.N., Wisniewski H., Halamka J.D., Kashavan M.S., Torous J.B. (2019). Chatbots and conversational agents in mental health: A review of the psychiatric landscape. Can. J. Psychiatry.

[15] Abd-Alrazaq A.A., Safi Z., Alajlani M., Warren J., Househ M., Denecke K. (2020). Technical metrics used to evaluate health care chatbots: Scoping review. J. Med. Internet Res..

[16] Torous J., Bucci S., Bell I.H., Kessing L.V., Faurholt-Jepsen M., Whelan P., Carvalho A.F., Keshavan M., Linardon J., Firth J. (2021). The growing field of digital psychiatry: Current evidence and the future of apps, social media, chatbots, and virtual reality. World Psychiatry.

[17] Hickey B.A., Chalmers T., Newton P., Lin C.T., Sibbritt D., McLachlan C.S., Clifton-Bligh R., Morley J., Lal S. (2021). Smart devices and wearable technologies to detect and monitor mental health conditions and stress: A systematic review. Sensors.

[18] Jobin A., Ienca M., Vayena E. (2019). The global landscape of AI ethics guidelines. Nat. Mach. Intell..

[19] Ghassemi M., Oakden-Rayner L., Beam A.L. (2021). The false hope of current approaches to explainable artificial intelligence in health care. Lancet Digit. Health.

[20] Vis C., Bührmann L., Riper H., Ossebaard H.C. (2020). Health technology assessment frameworks for eHealth: A systematic review. Int. J. Technol. Assess. Health Care.

[21] Liverpool S., Mc Donagh C., Feather J., Vrouva I., Law D. (2025). Updates on digital mental health interventions for children and young people: Systematic overview of reviews. Eur. Child Adolesc. Psychiatry.

[22] Khoo L.S., Kim H.K., Husain W. (2024). Machine learning for multimodal mental health detection: A systematic review of passive sensing approaches. Sensors.

[23] Ramanarayanan V. (2024). Multimodal technologies for remote assessment of neurological and mental health. J. Speech Lang. Hear. Res..

[24] Zhang Z., Zhang S., Ni D., Wei Z., Yang K., Jin S., Huang G., Liang Z., Zhang L., Li L. (2024). Multimodal sensing for depression risk detection: Integrating audio, video, and text data. Sensors.

[25] Khorev V., Kurkin S., Badarin A., Antipov V., Pitsik E., Andreev A., Grubov V., Drapkina O., Kiselev A., Hramov A. (2024). Review on the use of brain computer interface rehabilitation methods for treating mental and neurological conditions. J. Integr. Neurosci..

[26] Zhang Z., Chen Y., Zhao X., Fan W., Peng D., Li T., Zhao L., Fu Y. (2024). A review of ethical considerations for the medical applications of brain-computer interfaces. Cogn. Neurodyn..

[27] Fitzgerald P.J. (2024). Frontal alpha asymmetry and its modulation by monoaminergic neurotransmitters in depression. Clin. Psychopharmacol. Neurosci..

[28] Krishna D., Singh D., Manjunath N.K. (2025). Determining the depth of meditation through frontal alpha asymmetry. Front. Hum. Neurosci..

[29] Wang R., Zhu J., Wang S., Wang T., Huang J., Zhu X. (2024). Multi-modal emotion recognition using tensor decomposition fusion and self-supervised multi-tasking. Int. J. Multimed. Inf. Retr..

[30] Wang X.Q., Sun H.Q., Si J.Y., Lin Z.Y., Zhai X.M., Lu L. (2024). Challenges and suggestions of ethical review on clinical research involving brain-computer interfaces. Chin. Med. Sci. J..

[31] Farzan M., Ebrahimi H., Pourali M., Sabeti F. (2025). Artificial intelligence-powered cognitive behavioral therapy chatbots, a systematic review. Iran. J. Psychiatry.

[32] Cortes C., Vapnik V. (1995). Support-vector networks. Mach. Learn..

[33] Noble W.S. (2006). What is a support vector machine?. Nat. Biotechnol..

[34] Breiman L. (2001). Random forests. Mach. Learn..

[35] Biau G., Scornet E. (2016). A random forest guided tour. TEST.

[36] LeCun Y., Bengio Y., Hinton G. (2015). Deep learning. Nature.

[37] Krizhevsky A., Sutskever I., Hinton G.E. (2017). ImageNet classification with deep convolutional neural networks. Commun. ACM.

[38] Hochreiter S., Schmidhuber J. (1997). Long short-term memory. Neural Comput..

[39] Graves A., Mohamed A.R., Hinton G. Speech recognition with deep recurrent neural networks. Proceedings of the 2013 IEEE International Conference on Acoustics, Speech and Signal Processing.

[40] Vaswani A., Shazeer N., Parmar N., Uszkoreit J., Jones L., Gomez A.N., Kaiser Ł., Polosukhin I., Guyon I., Luxburg U.V., Bengio S., Wallach H., Fergus R., Vishwanathan S., Garnett R. (2017). Attention is all you need. Advances in Neural Information Processing Systems.

[41] Devlin J., Chang M.W., Lee K., Toutanova K. BERT: Pre-training of deep bidirectional transformers for language understanding. Proceedings of the 2019 Conference of the North American Chapter of the Association for Computational Linguistics: Human Language Technologies.

[42] Brown T., Mann B., Ryder N., Subbiah M., Kaplan J.D., Dhariwal P., Neelakantan A., Shyam P., Sastry G., Askell A., Larochelle H., Ranzato M., Hadsell R., Balcan M.F., Lin H. (2020). Language models are few-shot learners. Advances in Neural Information Processing Systems.

[43] Achiam J., Adler S., Agarwal S., Ahmad L., Akkaya I., Aleman F.L., Almeida D., Altenschmidt J., Altman S., Anadkat S. (2023). GPT-4 technical report. arXiv.

[44] Christiano P.F., Leike J., Brown T., Martic M., Legg S., Amodei D., Guyon I., Luxburg U.V., Bengio S., Wallach H., Fergus R., Vishwanathan S., Garnett R. (2017). Deep reinforcement learning from human preferences. Advances in Neural Information Processing Systems.

[45] Ouyang L., Wu J., Jiang X., Almeida D., Wainwright C.L., Mishkin P., Zhang C., Agarwal S., Slama K., Ray A., Koyejo S., Mohamed S., Agarwal A., Belgrave D., Cho K., Oh A. (2022). Training language models to follow instructions with human feedback. Advances in Neural Information Processing Systems.

[46] Bender E.M., Gebru T., McMillan-Major A., Shmitchell S. On the dangers of stochastic parrots: Can language models be too big?. Proceedings of the 2021 ACM Conference on Fairness, Accountability, and Transparency.

[47] Weidinger L., Mellor J., Rauh M., Griffin C., Uesato J., Huang P.S., Cheng M., Glaese M., Balle B., Kasirzadeh A. (2021). Ethical and social risks of harm from language models. arXiv.

[48] Liu Y., Wang A., Li Z., Sun M., Li L., Wang T., Liu H. (2025). A survey of embodied AI in healthcare: Techniques, applications, and opportunities. arXiv.

[49] Bilalpur M., Inan M., Zeinali D., Cohn J.F., Alikhani M. (2024). Learning to generate context-sensitive backchannel smiles for embodied AI agents with applications in mental health dialogues. arXiv.

[50] Liu Y., Wang S., Zhang L. (2025). Enhancing depression detection with chain-of-thought prompting: From emotion to reasoning using large language models. arXiv.

[51] Chen Y.X., Chang Y.C., Ho H.W. Empathy-enhanced chatbot for psychological support: A retrieval-augmented and therapy-informed approach. Proceedings of the 2025 IEEE 5th International Conference on Human-Machine Systems (ICHMS).

[52] Xu S., Yan Z., Dai C., Wu F. (2025). MEGA-RAG: A retrieval-augmented generation framework with multi-evidence guided answer refinement for mitigating hallucinations of LLMs in public health. Front. Public Health.

[53] Saleem K., Saleem M., Almogren A., Almogren A., Kaur U., Bharany S., Rehman A.U. (2025). Multi-agent based cognitive intelligence in non-linear mental healthcare-based situations. IEEE Access.

[54] Wu Y., Wan G., Li J., Zhao S., Ma L., Ye T., Pop I., Zhang Y., Chen J. (2025). ProAI: Proactive multi-agent conversational AI with structured knowledge base for psychiatric diagnosis. arXiv.

[55] Xu A., Yang D., Li R., Zhu J., Tan M., Yang M., Qiu W., Ma M., Wu H., Li B. (2025). AutoCBT: An autonomous multi-agent framework for cognitive behavioral therapy in psychological counseling. arXiv.

[56] Lee Y., Park S., Cho K., Bak J. (2024). MentalAgora: A gateway to advanced personalized care in mental health through multi-agent debating and attribute control. arXiv.

[57] Liu X., Zheng X., Cui W. (2024). Psychological counseling with integration of knowledge graph and multi-agent collaboration. J. Shanghai Jiaotong Univ. (Sci.).

[58] Zhang Y., Yang X., Li X., Yu S., Luan Y., Feng S., Wang D., Zhang Y. (2024). PsyDraw: A multi-agent multimodal system for mental health screening in left-behind children. arXiv.

[59] Chen K., Li X., Yang T., Wang H., Dong W., Gao Y. (2025). MDTeamGPT: A self-evolving LLM-based multi-agent framework for multi-disciplinary team medical consultation. arXiv.

[60] Kampman O.P., Phang Y.S., Han S., Xing M., Hong X., Hoosainsah H., Tan C., Winata G.I., Wang S., Heaukulani C. (2024). A multi-agent dual dialogue system to support mental health care providers. arXiv.

[61] Heinz M.V., Mackin D.M., Trudeau B.M., Bhattacharya S., Wang Y., Banta H.A., Jewett A.D., Salzhauer A.J., Griffin T.Z., Jacobson N.C. (2025). Randomized trial of a generative AI chatbot for mental health treatment. NEJM AI.

[62] Na H. (2024). CBT-LLM: A Chinese large language model for cognitive behavioral therapy-based mental health question answering. arXiv.

[63] Wang S., Jiang S., Gao Y., Wang B., Gao S., Zhuang X. (2025). Empowering medical multi-agents with clinical consultation flow for dynamic diagnosis. arXiv.

[64] Ji S., Zheng X., Sun J., Chen R., Gao W., Srivastava M. (2024). Mindguard: Towards accessible and stigma-free mental health first aid via edge LLM. arXiv.

[65] Sin J. (2024). An AI chatbot for talking therapy referrals. Nat. Med..

[66] Chaudhry B.M., Debi H.R. (2024). User perceptions and experiences of an AI-driven conversational agent for mental health support. mHealth.

[67] Rahsepar Meadi M., Sillekens T., Metselaar S., van Balkom A., Bernstein J., Batelaan N. (2025). Exploring the ethical challenges of conversational AI in mental health care: Scoping review. JMIR Ment. Health.

[68] Saeidnia H.R., Hashemi Fotami S.G., Lund B., Ghiasi N. (2024). Ethical considerations in artificial intelligence interventions for mental health and well-being: Ensuring responsible implementation and impact. Soc. Sci..

[69] Ratwani R.M., Sutton K., Galarraga J.E. (2024). Addressing AI algorithmic bias in health care. JAMA.

[70] Tavory T. (2024). Regulating AI in mental health: Ethics of care perspective. JMIR Ment. Health.

[71] World Health Organization (2021). Ethics and Governance of Artificial Intelligence for Health: WHO Guidance.

[72] Madiega T. (2021). Artificial Intelligence Act.

[73] Arrieta A.B., Díaz-Rodríguez N., Del Ser J., Bennetot A., Tabik S., Barbado A., García S., Gil-López S., Molina D., Benjamins R. (2020). Explainable Artificial Intelligence (XAI): Concepts, taxonomies, opportunities and challenges toward responsible AI. Inf. Fusion.

[74] Tjoa E., Guan C. (2021). A survey on explainable artificial intelligence (XAI): Toward medical XAI. IEEE Trans. Neural Netw. Learn. Syst..

[75] Lundberg S.M., Lee S.I., Guyon I., Luxburg U.V., Bengio S., Wallach H., Fergus R., Vishwanathan S., Garnett R. (2017). A unified approach to interpreting model predictions. Advances in Neural Information Processing Systems.

[76] Ribeiro M.T., Singh S., Guestrin C. “Why should I trust you?”: Explaining the predictions of any classifier. Proceedings of the 22nd ACM SIGKDD International Conference on Knowledge Discovery and Data Mining.

[77] Friedman B., Hendry D.G. (2019). Value Sensitive Design: Shaping Technology with Moral Imagination.

[78] Umbrello S., De Bellis A.F., Roman V. (2018). A value-sensitive design approach to intelligent agents. Artificial Intelligence Safety and Security.

[79] Gilbert S., Fenech M., Hirsch M., Upadhyay S., Biasiucci A., Starlinger J. (2021). Algorithm change protocols in the regulation of adaptive AI. J. Med. Internet Res..

[80] U.S. Food and Drug Administration (2021). Artificial Intelligence/Machine Learning (AI/ML)-Based Software as a Medical Device (SaMD) Action Plan.

[81] Babu A., Joseph A.P. (2024). Artificial intelligence in mental healthcare: Transformative potential vs. the necessity of human interaction. Front. Psychol..

[82] Kuhail M.A., Alturki N., Thomas J., Alkhalifa A.K., Alshardan A. (2025). Human-human vs human-AI therapy: An empirical study. Int. J. Hum.-Comput. Interact..

[83] Xiong F., Yu X., Leong H.W., Ma A. (2025). AI-driven research ecosystem: Unifying human-AI collaboration models and new research thinking paradigms. J. Educ. Technol. Innov..

[84] AlMakinah R., Norcini-Pala A., Disney L., Canbaz M.A. Enhancing mental health support through human-AI collaboration: Toward secure and empathetic AI-enabled chatbots. Proceedings of the 2025 IEEE Conference on Artificial Intelligence (CAI).

[85] Yoo D.W., Woo H., Pendse S.R., Lu N.Y., Birnbaum M.L., Abowd G.D., De Choudhury M. (2024). Missed opportunities for human-centered AI research: Understanding stakeholder collaboration in mental health AI research. Proc. ACM Hum.-Comput. Interact..

[86] Zhang F., Chen Z. (2024). A novel reinforcement learning-based particle swarm optimization algorithm for better symmetry between convergence speed and diversity. Symmetry.

[87] American Psychological Association Study Demonstrates Utility of GenAI Chatbot for Treating Mental Health Conditions. https://www.apaservices.org/practice/business/technology/on-the-horizon/mental-health-chatbot.

[88] Raffaelli R., Tushman M.L. (2025). Crisis at Chai AI; Harvard Business School Case 762-b62.

[89] Coeckelbergh M. (2023). Chatbots Can Kill: The Suicide of a Belgian Man Raises Ethical Issues about the Use of ChatGPT. Medium.

[90] Yeung J.A., Dalmasso J., Foschini L., Dobson R.J., Kraljevic Z. (2025). The psychogenic machine: Simulating AI psychosis, delusion reinforcement and harm enablement in large language models. arXiv.

[91] Clegg K.A. (2025). Shoggoths, sycophancy, psychosis, oh my: Rethinking large language model use and safety. JMIR Ment. Health.

[92] Shen Y., Liang J., Kang H., Sun X., Chen Q. (2025). NLAPSMjSO-EDA: A nonlinear shrinking population strategy algorithm for elite group exploration with symmetry applications. Symmetry.

[93] Tricco A.C., Lillie E., Zarin W., O’Brien K.K., Colquhoun H., Levac D., Moher D., Peters M.D., Horsley T., Weeks L. (2018). PRISMA extension for scoping reviews (PRISMA-ScR): Checklist and explanation. Ann. Intern. Med..

[94] Ma A., Chen J., Yang Z. From Tool to Agent: Review of AI’s Paradigm Shift and Proposal for a Tiered Human-AI Healing Ecosystem in Mental Health. Proceedings of the 2025 International Conference on Intelligent Education and Intelligent Research (IEIR 2025).

