# Supplementary material for: From Tool to Agent: A Semi-Systematic Review of Human–AI Alignment and a Proposed Tiered Healing Ecosystem for Mental Health
Source: Healthcare (Basel). 2026 Mar 23;14(6):820. doi: 10.3390/healthcare14060820 (PMC13026511; doi:10.3390/healthcare14060820)
Supplement: Supplementary file 1 [file healthcare-14-00820-s001.zip › File_ S1_Detailed Search.pdf]

Appendix B: Detailed Search Strategy

To ensure the utmost transparency and reproducibility of the literature screening process for this semi-systematic review, the specific Boolean search strings, database-specific syntax, applied filters, and execution dates are thoroughly documented below.

Given the interdisciplinary nature of the proposed Tiered Human-AI Healing Ecosystem (THHE)—which intersects computer science, human-computer interaction (HCI), and clinical psychology—we purposefully selected three distinct databases: PubMed (for clinical and psychiatric literature), IEEE Xplore (for technical and engineering literature), and the ACM Digital Library (for HCI and computational system literature).

All systematic searches were executed on October 15, 2025.

1. PubMed Search Strategy

- **Execution Date:** October 15, 2025
- **Applied Filters:** English Language; Publication Date from 01/01/2020 to 10/15/2025; Peer-reviewed Journal Articles.
- **Database-Specific Syntax:** [MeSH] (Medical Subject Headings), [tiab] (Title/Abstract).
- **Complete Boolean String:**

(("Artificial Intelligence"[MeSH] OR "Generative AI"[tiab] OR "Large Language Models"[tiab] OR "LLM"[tiab] OR "Conversational Agent"[tiab] OR "Chatbot"[tiab] OR "Virtual Agent"[tiab]) AND ("Mental Health"[MeSH] OR "Psychiatry"[MeSH] OR "Psychotherapy"[MeSH] OR "Cognitive Behavioral Therapy"[tiab] OR "Therapeutic Alliance"[tiab] OR "Digital Intervention"[tiab]))

- **Initial Hits:** 1050

2. IEEE Xplore Search Strategy

- **Execution Date:** October 15, 2025
- **Applied Filters:** Publisher: IEEE; Content Type: Journals & Conferences; Year: 2020–2025.
- **Database-Specific Syntax:** Metadata search focusing on Document Title and Abstract.
- **Complete Boolean String:**

((("Document Title": "Artificial Intelligence" OR "Abstract": "Artificial Intelligence" OR

"Document Title": "Large Language Model" OR "Abstract": "Large Language Model" OR "Document Title": "Chatbot" OR "Abstract": "Chatbot" OR "Document Title": "Conversational Agent" OR "Abstract": "Conversational Agent") AND ("Document Title": "Mental Health" OR "Abstract": "Mental Health" OR "Document Title": "Psychology" OR "Abstract": "Psychology" OR "Document Title": "Affective Computing" OR "Abstract": "Affective Computing" OR "Document Title": "Well-being" OR "Abstract": "Well-being")))

- **Initial Hits:** 820

3. ACM Digital Library Search Strategy

- **Execution Date:** October 15, 2025
- **Applied Filters:** Published since 2020; Research Articles.
- **Database-Specific Syntax:** Title: and Abstract: tags.
- **Complete Boolean String:**

((Title:("Artificial Intelligence" OR "Large Language Model" OR "Chatbot" OR "Virtual Agent" OR "Conversational AI") OR Abstract:("Artificial Intelligence" OR "Large Language Model" OR "Chatbot" OR "Virtual Agent" OR "Conversational AI")) AND (Title:("Mental Health" OR "Therapy" OR "Counseling" OR "Psychiatry" OR "Psychological support") OR Abstract:("Mental Health" OR "Therapy" OR "Counseling" OR "Psychiatry" OR "Psychological support")))

- **Initial Hits:** 580

Search Strategy Note: The initial records retrieved across the three databases (totaling 2,450 records) were exported to a reference management software where duplicates were automatically and manually removed prior to the title/abstract screening phase outlined in the PRISMA flow diagram.
