# Supplementary material for: From Tool to Agent: A Semi-Systematic Review of Human–AI Alignment and a Proposed Tiered Healing Ecosystem for Mental Health
Source: Healthcare (Basel). 2026 Mar 23;14(6):820. doi: 10.3390/healthcare14060820 (PMC13026511; doi:10.3390/healthcare14060820)
Supplement: Supplementary file 1 [file healthcare-14-00820-s001.zip › Table_S2 updated.pdf]

Appendix A: Classification of Cited References

To ensure methodological transparency and conceptual clarity, all cited references (N=93) have been systematically categorized based on their role in this review. This classification distinguishes the 61 included studies (the evidence base for the AI-T and AI-A paradigms) from foundational technical reports and general medical AI background.

Table S2. Categorization of References by Paradigm and Evidence Type

Category (Domain)	Scope & Role in the Review	Reference Numbers (N=93)	Core Sub-total (n=61)
**I. Empirical Mental Health AI Research**	Randomized Controlled Trials (RCTs), pilot studies, and user perception studies directly evaluating AI in therapeutic contexts (e.g., Therabot, AutoCBT).	[13], [24], [27–29], [49–52], [61–64], [65–66], [82], [85], [90]	**18**
**II. Clinical Reviews & Scoping Surveys**	Systematic and scoping reviews focusing on chatbots, digital biomarkers, and LLMs specifically applied to mental healthcare.	[1–6], [8–9], [14–17], [20–23], [25], [31], [48], [67], [73–74], [93]	**23**
**III. Frontier Architectures & Case Studies**	Novel AI frameworks (e.g., Multi-Agent Systems, RAG) and critical ethical case reports (e.g., Harvard case study, risk incidents) that inform the AI-A paradigm.	[43], [53–60], [70–72], [81], [83–84], [87–89], [91–92]	**20**
**IV. Foundational Theory & Methodology**	General AI algorithms (e.g., SVM, Transformer), non-psychiatric medical AI background, and broad policy documents.	[7], [10–12], [18–19], [26], [30], [32–42], [44–47], [68–69], [75–80], [86]	*(Excluded from PRISMA n=61)*
**Total**		**N = 93**	**Total Included: 61**

Quality Appraisal of Included Studies (n=61) Based on the principles of the Mixed Methods Appraisal Tool (MMAT) adapted for this semi-systematic review, the 61 core studies were evaluated for empirical robustness and study design. The specific categorization of the references is detailed below:

- **High Quality (n=15):** Rigorous Randomized Controlled Trials (RCTs), large-scale empirical studies, and high-quality systematic reviews. **References:** [1, 2, 8, 13, 14,

20, 24, 27, 28, 50, 51, 62, 74, 85, 90]

- **Moderate Quality (n=34):** Pilot studies, user perception studies with smaller cohorts, scoping reviews, and partially validated architectural frameworks. **References:** [3, 4, 5, 6, 9, 15, 16, 17, 21, 22, 23, 25, 29, 31, 48, 49, 52, 53, 54, 55, 56, 57, 58, 59, 60, 61, 63, 64, 65, 66, 67, 73, 82, 93]
- **Low Quality (n=12):** Purely conceptual proposals, early-stage architectures lacking human-subject data, and isolated ethical case reports. **References:** [43, 70, 71, 72, 81, 83, 84, 87, 88, 89, 91, 92]
